# Examining mental health knowledge, stigma, and service use intentions among Royal Canadian Mounted Police cadets

**DOI:** 10.3389/fpsyg.2023.1123361

**Published:** 2023-05-02

**Authors:** Katie L. Andrews, Laleh Jamshidi, Robyn E. Shields, Taylor A. Teckchandani, Tracie O. Afifi, Amber J. Fletcher, Shannon Sauer-Zavala, Alain Brunet, Gregory P. Krätzig, R. Nicholas Carleton

**Affiliations:** ^1^Canadian Institute of Public Safety Research and Treatment (CIPSRT), University of Regina, Regina, SK, Canada; ^2^Department of Community Health Sciences, University of Manitoba, Winnipeg, MB, Canada; ^3^Department of Sociology and Social Studies, University of Regina, Regina, SK, Canada; ^4^Treatment Innovation for Psychological Services Research Program, Department of Psychology, University of Kentucky, Lexington, KY, United States; ^5^McGill’s Psychiatry Department and Douglas Institute Research Center, Verdun, QC, Canada; ^6^Anxiety and Illness Behaviours Laboratory, Department of Psychology, University of Regina, Regina, SK, Canada

**Keywords:** mental health training, public safety personnel, police, mental health services, help-seeking behavior, mental health stigma, cadets

## Abstract

**Background:**

Royal Canadian Mounted Police (RCMP) officers experience an elevated risk for mental health disorders due to inherent work-related exposures to potentially psychologically traumatic events and occupational stressors. RCMP officers also report high levels of stigma and low levels of intentions to seek mental health services. In contrast, very little is known about the levels of mental health knowledge and stigma of RCMP cadets starting the Cadet Training Program (CTP). The current study was designed to: (1) obtain baseline levels of mental health knowledge, stigma against peers in the workplace, and service use intentions in RCMP cadets; (2) determine the relationship among mental health knowledge, stigma against peers in the workplace, and service use intentions among RCMP cadets; (3) examine differences across sociodemographic characteristics; and (4) compare cadets to a sample of previously surveyed serving RCMP.

**Methods:**

Participants were RCMP cadets (*n* = 772) starting the 26-week CTP. Cadets completed questionnaires assessing mental health knowledge, stigma against coworkers with mental health challenges, and mental health service use intentions.

**Results:**

RCMP cadets reported statistically significantly lower levels of mental health knowledge (*d* = 0.233) and stigma (*d* = 0.127), and higher service use intentions (*d* = 0.148) than serving RCMP (all *p*s < 0.001). Female cadets reported statistically significantly higher scores on mental health knowledge and service use and lower scores on stigma compared to male cadets. Mental health knowledge and service use intentions were statistically significantly positively associated. For the total sample, stigma was inversely statistically significantly associated with mental health knowledge and service use intentions.

**Conclusion:**

The current results indicate that higher levels of mental health knowledge were associated with lower stigma and higher intention to use professional mental health services. Differences between cadets and serving RCMP highlight the need for regular ongoing training starting from the CTP, designed to reduce stigma and increase mental health knowledge. Differences between male and female cadets suggest differential barriers to help-seeking behaviors. The current results provide a baseline to monitor cadet mental health knowledge and service use intentions and stigma as they progress throughout their careers.

## 1. Introduction

Public safety personnel [PSP; Canadian Institute for Public Safety Research and Treatment (CIPSRT), 2019] include diverse professionals (e.g., border services personnel, correctional workers, firefighters, operational and intelligence personnel, paramedics, police, public safety communicators, search, and rescue personnel) regularly engaged in maintaining public safety [Canadian Institute for Public Safety Research and Treatment (CIPSRT), 2019; Public Safety Canada, 2019]. PSP are frequently exposed to potentially psychologically traumatic events (PPTEs; Carleton et al., 2019) and other occupational stressors (Carleton et al., 2020a), collectively increasing their risk for developing posttraumatic stress injuries [PTSI; Canadian Institute for Public Safety Research and Treatment (CIPSRT), 2019; Public Safety Canada, 2019]. In Canada, Royal Canadian Mounted Police (RCMP) report very frequent exposures to diverse PPTEs (Carleton et al., 2019), which can compromise the physical (Violanti, 2006; Levy-Gigi et al., 2014; Violanti, 2015), psychological (Skogstad et al., 2013; Klimley et al., 2018; Sherwood et al., 2019), and relational (Paton et al., 1999; Woody, 2006; Kirschman et al., 2015) health of police officers. Initial evidence indicates serving RCMP report high proportions of positive screenings for mental health disorders with 50.2% screening positive for one or more mental health disorders (Carleton et al., 2018a). This is much higher than the diagnostic prevalence of the general population (10.1%; Statistics Canada, 2014). The reported high prevalence of mental disorder symptoms by serving RCMP is coupled with reported high levels of stigma and low levels of intentions to seek mental health services (Krakauer et al., 2020), suggesting a problematic confluence of serious mental health challenges for serving RCMP.

The frequent PPTE exposures and associated high levels of reported mental health symptoms among PSP have led to substantial efforts designed to increase mental health education and treatment. Several strategies and programs have been developed among PSP to minimize and manage the impact of PPTEs. Mental health training has included programs such as the Road to Mental Readiness (R2MR; Carleton et al., 2018b; Szeto et al., 2019), critical incident stress management/debriefing (CISM/CISD), mental health first aid (MHFA; Hadlaczky et al., 2014), Before Operational Stress (BOS; WGM Psychological Services, 2018), and peer support. The mental health training is intended to reduce mental disorder prevalence by increasing knowledge, reducing stigma, and therein promoting early interventions. The available cross-sectional evidence suggests that participation in any of the listed mental health training programs can be associated with greater willingness to access support and reduction in the likeliness of screening positive for a mental disorder (Carleton et al., 2019). Interventions appear to yield statistically significant short-term benefits in terms of formal help-seeking, self-help, mental health literacy, and personal stigma, as well as positive long-term effects on formal help-seeking behaviors (Xu et al., 2018). Higher levels of mental health knowledge have also been associated with lower levels of stigma and higher levels of service use intentions in both in the general public (Hadlaczky et al., 2014; Schnyder et al., 2017; Xu et al., 2018) and among PSP (Krakauer et al., 2020).

The high levels of stigma and low levels of reported intentions to seek mental health services among RCMP suggest mental health training and resources may help to increase individual abilities to identify and manage mental health challenges, recognize when a disorder may be developing, and identify evidence-based options for help-seeking or treatment (Reavley and Jorm, 2011; Jorm, 2012). Increased mental health knowledge can also reduce levels of associated stigma and negative attitudes, which have been inversely associated with symptom recognition and help-seeking behaviors (Corrigan, 2004; Conner et al., 2010; Clement et al., 2015; Cheng et al., 2018). RCMP may avoid accessing services for fear of retribution by peers or administration (Blum, 2000; Karaffa and Koch, 2016; White et al., 2016; Wheeler et al., 2021). RCMP may also feel uncomfortable disclosing mental health challenges for fear of being perceived as unfit for duty or weak (Newell et al., 2022), which could further reduce their social supports and treatment-seeking behaviors (Violanti, 2010; Wilmoth, 2014). Lower levels of mental health knowledge and increased stigma appear to be current barriers for reducing mental health challenges among serving RCMP.

The RCMP has prioritized deploying diverse solutions to mitigate mental health challenges facing RCMP members (Public Safety Canada, 2019); however, the many proffered programs to support PSP mental health have very little research evidence regarding their effectiveness. There have been few evaluations of mental health training programs for PSP (Beshai and Carleton, 2016; Maslowski et al., 2019; Szeto et al., 2019; Anderson et al., 2020; Di Nota et al., 2021; Price et al., 2022). A recent systematic review of the available literature observed high variability in study design, target audience, duration of training, time or interventions, outcomes measured, and timing of follow-up across the programs and associated evaluation (Anderson et al., 2020); accordingly, comparing the effectiveness of the programs is extremely difficult and quality assessments of the impact of such program on the mental health of PSP is rarely available (Anderson et al., 2020).

The extant programs associated with research have produced positive effects. For example, Canadian firefighters and paramedics perceived critical incident stress management (CISM) as a beneficial and valuable tool for providing skills and coping strategies and offered some mental health benefits for symptoms of AUD and GAD when delivered with high fidelity (Price et al., 2022). Mental Health First Aid (MHFA) was observed to produce moderate improvements in mental health knowledge and confidence of trainees to help those in need (Maslowski et al., 2019). Road to Mental Readiness (R2MR) produced favorable results for a group of PSP (Szeto et al., 2019; Anderson et al., 2020) and paramedic students (Vaughan et al., 2020), including improvements in mental health outcomes and stigma and increasing resiliency skills, although effects were time-limited with rapid skill decay. Before Operational Stress (BOS) produced small improvements in PTSD symptoms, quality of life, perceived social support, and stigma from baseline to 6 months later. The available evidence suggests that mental health training programs may be effective at improving mental health knowledge and reducing stigma; however, a great deal of heterogeneity was observed across the evaluation methods in the studies leading to substantial difficulties when determining which mental health training program as most effective.

The RCMP is working to address the existing limitations through the RCMP Study (Carleton et al., 2022) by developing, deploying, and longitudinally assessing a multi-modal mental health solution that includes evidence-based biopsychosocial assessments and evidence-informed integrated cadet mental health training. The RCMP Study can also help to address the limited research evidence regarding the mental health knowledge, stigma, and service use intentions of RCMP cadets starting their careers. The high prevalence of mental health disorder symptoms, high levels of stigma, and low levels of intentions to seek mental health services may be related to years of service or to limited mental health training.

The current study examines mental health knowledge, stigma against peers in the workplace with mental health challenges, and mental health service use intentions of RCMP cadets prior to starting the Cadet Training Program (CTP). The current study was designed to: (1) obtain baseline levels of mental health knowledge, stigma against peers in the workplace, and service use intentions of RCMP cadets; (2) determine the relationship among mental health knowledge, stigma against peers in the workplace, and service use intentions among RCMP cadets; and (3) examine differences across sociodemographic characteristics; and (4) compare cadets to a sample of previously surveyed serving RCMP. Mental health knowledge was expected to be positively associated with service use intentions and inversely associated with stigma. Based on previous research, it was hypothesized that male cadets would report greater mental health stigma than their female counterparts (Soomro and Yanos, 2019) and female cadets would report higher mental health knowledge and service use intentions than male cadets (Krakauer et al., 2020).

## 2. Materials and methods

### 2.1. Procedure

Data were collected using a web-based self-report survey in English or French as part of the RCMP study. Full details on the RCMP Study have been published in a dedicated protocol paper (Carleton et al., 2022). The study was approved by the University of Regina Institutional Research Ethics Board (file No. 2019-055) and the RCMP Research Ethics Board (file No. SKM_C30818021312580). The study was also approved through a Privacy Impact Assessment as part of the overall approval NARMS 201611123286 and PSPC 201701491/M7594174191. The current study focused on cross-sectional data collected from the Full Assessment administered when participants started the CTP (i.e., pre-training) between May 2019 and October 2021. During the Full Assessment, cadets self-reported outcomes on mental health knowledge, stigma in the workplace, and mental health service use intentions.

### 2.2. Data and sample

Participants were RCMP cadets (*n* = 772) starting the 26-week CTP. To qualify for CTP, cadets must be Canadian citizens or permanent residents, 19–57 years old, who can fluently read, write, and speak either English or French (Hembroff and Krätzig, 2020). Cadets must also meet several recruiting requirements, including security clearances, medical examinations, a polygraph test, and minimum physical standards. There were no conditions excluding persons otherwise qualified for the CTP. A total of 1,696 cadets were invited to participate in the RCMP Study as part of the standard training condition (Carleton et al., 2022). The final sample was a total of 772 cadets. Most participants (98%; *n* = 759) completed all of the survey questions associated with the current analyses. Cadets were predominantly male (72.3%), aged 19–29 years old (60.1%). Considerable proportions of participants were single (46.9%) or married or in common-law relationships (i.e., living with a person in a conjugal relationship for 12 continuous months) (43.1%), residing in Western Canada (52.8%) or Eastern Canada (34.5%), with no pervious PSP or Canadian Armed Forces (CAF) experience (56.8%).

### 2.3. Self-report measures

#### 2.3.1. Mental Health Knowledge Scale

The Mental Health Knowledge Scale (MAKS; Evans-Lacko et al., 2010) is a 12-item self-report questionnaire. The first six items assess beliefs about mental health (e.g., “People with severe mental health problems can fully recover”) proceeded by nine items designed to assess participants’ level of recognition and familiarity with various mental health conditions (e.g., stress; posttraumatic stress disorder). All items were rated on a 5-point Likert scale ranging from 1 (strongly disagree) to 5 (strongly agree). Data support the test–retest reliability (Evans-Lacko et al., 2010) and the Cronbach’s *α* was *α* = 0.39 for the current sample.

#### 2.3.2. Open Minds Survey for Workplace Attitudes

The Open Minds Survey for Workplace Attitudes-Short Form (OMS-WA-SF; Boehme et al., 2022) is a self-report questionnaire that includes 9 items designed to measure Attitudes Predicting Avoidance and Beliefs about Danger/Unpredictability toward people with mental health challenges. Items such as “I would try to avoid a co-worker with a mental illness” were rated on a 5-point Likert scale ranging from 1 (strongly disagree) to 5 (strongly agree). Higher scores indicate higher mental health stigma in the workplace. The Mental Health Commission of Canada employs the OMS-WA as a standard metric for stigma. The OMS-WA had a Cronbach’s *α* = 0.87 in the total sample.

#### 2.3.3. Mental Health Service Use Questionnaire

Mental Health Service Use Questionnaire (MHSUQ) is a 4-item self-report questionnaire designed to measure willingness to seek professional help for mental health challenges. Items (e.g., “If I developed mental health problems, I would want to seek mental health treatment from a professional”) are rated on a 7-point Likert scale, ranging from 1 (strongly disagree) to 7 (strongly agree). The MHSUQ is derived from the 76-item CAF-R-MHSUQ (Fikretoglu et al., 2019) and is consistent with questions regularly used in Statistics Canada surveys to assess mental health service use. The Cronbach’s *α* for the MHSUQ was *α* = 0.95 in the current sample.

### 2.4. Statistical analyses

Frequencies and percentages of sociodemographic information including sex, age, marital status, province of residence, education, and previous PSP or CAF experience were described. The means and standard deviations of mental health knowledge, stigma, and service use intention scores were calculated across different sociodemographic groups. A series of independent sample *t*-tests and one-way analyses of variances (ANOVA) were conducted to assess differences in means of mental health knowledge, stigma, and service use intention scores across different sociodemographic categories, as well as the entire sample and previously published serving RCMP (Krakauer et al., 2020) collected using similar methods as the current study. Statistically significant differences between sociodemographic categories were evaluated through post-hoc analyses using Holm-Bonferroni adjustments for multiple comparisons to control Type I errors. Zero-order correlations were performed between mental health knowledge, stigma, and service use intention scores within the entire sample to evaluate the variable interrelationships. Multivariate analysis of variance (MANOVA) was performed to assess whether there were significant differences on a linear combination of mental health knowledge, stigma, and service use intention scores across sociodemographic categories. Statistical significance level was set at *p* ≤ 0.05. All data were analyzed using SPSS v.28 Premium (IBM, 2021 New York, United States).

## 3. Results

The sociodemographic variables and mean scores of mental health knowledge, stigma, and service use intention across demographic categories are provided in Table 1. Some sociodemographic and sex differences were observed. Females reported statistically significantly higher mental health knowledge (*p* < 0.05) and service use intention scores (*p* < 0.05) and lower stigma scores (*p* < 0.001). A statistically significant effect of age (*p* < 0.05), ethnicity (*p* < 0.05), and province of residence (*p* < 0.05) on mental health knowledge scores were observed; however, follow-up multiple pairwise comparisons were not statistically significant due to application of Holm-Bonferroni adjustment to control familywise error rates. Individuals from Atlantic Canada reported statistically significantly lower stigma scores (*p* < 0.01) than cadets from Western Canada. Asian cadets reported statistically significantly higher stigma scores (*p* < 0.01) than First Nation/Inuit/Metis and White/Caucasian cadets. Cadets with high school education or less reported statistically significantly higher stigma scores (*p* < 0.01) than those with university degree education.

**Table 1:** Sociodemographic characteristics and comparison of MAKS, OMS-WA, and MHSUQ scores across different categories.

	% (*n*)^1^	MAKS	OMS-WA	MHSUQ
		Mean (SD)	Mean (SD)	Mean (SD)
Sex
Male	72.3 (549)	46.01 (4.32)	16.40 (5.53)	23.48 (5.22)
Female	24.8 (188)	46.81 (4.02)	14.48 (5.22)	24.35 (4.68)
Effect Size (Cohen’s *d*)	–	0.188*	0.351***	0.171*
Age
19–29	60.1 (456)	46.58 (4.14)	15.73 (5.62)	23.45 (5.00)
30–39	27.9 (212)	45.50 (4.36)	15.96 (5.07)	23.84 (5.32)
40–49	6.2 (47)	46.00 (4.89)	17.13 (5.98)	24.57 (5.28)
50–59	0.7 (5)	47.40 (2.97)	19.60 (5.59)	26.80 (2.68)
60 and older	–	–	–	–
Effect size ( $\eta_{p}^{2}$ )	–	0.014*	0.007	0.006
Marital status
Single	46.9 (356)	46.27 (4.22)	15.97 (5.67)	23.42 (5.19)
Separated/Divorced	1.4 (11)	47.82 (2.36)	14.18 (5.21)	26.36 (2.20)
Married/Common-Law	43.1 (327)	45.98 (4.41)	15.98 (5.44)	23.87 (5.04)
Widowed	–	–	–	–
Effect size ( $\eta_{p}^{2}$ )	–	0.004	0.002	0.006
Ethnicity
Asian	5.8 (44)	45.02 (3.36)	18.80 (6.32)^b^	24.25 (4.56)
Black	3.2 (24)	45.58 (3.28)	15.33 (6.38)^a,b^	23.92 (4.95)
First Nation/Inuit/Metis	3.0 (23)	46.96 (4.43)	14.00 (4.60)^a^	24.35 (5.01)
Hispanic	1.4 (11)	43.27 (4.08)	17.55 (6.93)^a,b^	27.36 (1.57)
South Asian	5.8 (44)	46.02 (4.92)	15.77 (5.27)^a,b^	24.18 (5.77)
White/Caucasian	73.1 (555)	46.44 (4.30)	15.81 (5.40)^a^	23.47 (5.18)
Effect size ( $\eta_{p}^{2}$ )	–	0.016*	0.023**	0.011
Province of residence
Western Canada (BC, AB, SK, MB)	52.8 (401)	46.04 (4.40)	16.37 (5.54)^a^	23.77 (5.14)
Eastern Canada (ON, QC)	34.5 (262)	46.11 (4.04)	15.84 (5.40)^a,b^	23.79 (4.85)
Atlantic Canada (PEI, NS, NB, NFL)	11.3 (86)	47.43 (4.31)	14.06 (5.35)^b^	23.35 (5.56)
Northern Territories (YK, NWT, NVT)	1.1 (8)	45.25 (1.91)	16.25 (5.57)^a,b^	25.25 (3.24)
Effect size ( $\eta_{p}^{2}$ )	–	0.011*	0.017**	0.002
Education		
High school graduate or less	10.3 (78)	45.72 (4.50)	17.58 (5.40)^a^	23.05 (5.55)
Some post-secondary school	43.2 (328)	46.01 (4.21)	15.96 (5.39)^a,b^	23.62 (5.12)
University degree/4-year college or higher	39.5 (300)	46.61 (4.21)	15.36 (5.39)^b^	24.10 (4.91)
Effect size ( $\eta_{p}^{2}$ )	–	0.006	0.015**	0.004
Previous PSP or military experience		
Yes	30.4 (231)	46.05 (4.61)	16.14 (5.66)	24.06 (4.78)
No	56.8 (431)	46.36 (4.21)	15.74 (5.47)	23.54 (4.78)
Effect size (Cohen’s *d*)	–	0.073	0.072	0.102
Total sample	100 (759)	46.2 (4.28)	15.94 (5.51)	23.73 (5.07)

MAKS, Mental Health Knowledge Schedule; OMS-WA, Open Minds Survey for Workplace Attitudes; MHSUQ, Mental Health Service Use Questionnaire. ^*^p < 0.05, ^**^p < 0.01, ^***^p < 0.001. Statistically significantly different; Holm-Bonferroni adjustment was applied to alpha level to control type I error in multiple comparisons. *M* (SD) represents Mean (Standard Deviation). $\eta_{p}^{2}$ represents partial Eta Square. Lettered superscripts within each column category indicate significant differences between category groups on respective measure at *p* ≤ 0.05. Means followed by a common letter are not significantly different.

1 Total percentages may not sum to 100 and ns may not sum to 759 due to non-response or responding “other.”

Mental health knowledge and service use intentions scores were statistically significantly correlated (*r* = 0.119, *p* < 0.01). The MANOVA indicated statistically significant differences between sex categories (*F*(3, 733) = 6.70, *p* < 0.001, Wilks’ Lambda = 0.973), age categories (*F*(9, 1738) = 2.49, *p* < 0.01, Wilks’ Lambda = 0.969), ethnicity categories (*F*(15, 1913) = 2.39, *p* < 0.01, Wilks’ Lambda = 0.950), province of residence (*F*(9, 1828) = 2.25, *p* < 0.05, Wilks’ Lambda = 0.974), and education levels (*F*(6, 1,402) = 2.36, *p* < 0.05, Wilks’ Lambda = 0.980) on a linear combination of mental health knowledge, stigma, and service use intention scores. For the cadets, stigma was inversely statistically significantly correlated with mental health knowledge (*r* = −0.268, *p* < 0.001) and service use intentions (*r* = −0.177, *p* < 0.001).

Comparisons between cadets and previously published data from serving RCMP on mental health knowledge, stigma, and service use intention scores are presented in Table 2. Current RCMP cadets reported statistically significantly lower mental health knowledge (*d* = 0.233, *p* < 0.001) and stigma scores (*d* = 0.127, *p* < 0.01), and higher services use intention scores (*d* = 0.148, *p* < 0.001) compared to serving RCMP.

**Table 2:** MAKS, OMS-WA and MHSUQ scores and descriptive statistics based on current and previously published self-report measures.

	Current RCMP cadets	Serving RCMP^1^	Comparing current RCMP cadets and serving RCMP
	Mean (SD)	Mean (SD)	Effect size (*d*)
MAKS	46.20 (4.28)	47.20 (4.33)	0.233***
OMSWA	15.94 (5.51)	16.67 (5.91)	0.127**
MHSUQ	23.73 (5.07)	22.88 (6.21)	0.148***
Total sample	759	1,070	–

MAKS, Mental Health Knowledge Schedule; OMS-WA, Open Minds Survey for Workplace Attitudes; MHSUQ, Mental Health Service Use Questionnaire; SD, standard deviation; RCMP, Royal Canadian Mounted Police. ^**^p < 0.01, ^***^p < 0.001. Statistically significantly different.

1 Krakauer et al. (2020); OMSWA scores was calculated based on 9-item scale used in the current study, other than 11-item version used in Krakauer et al. (2020).

## 4. Discussion

The current results help to fill important gaps in the literature on RCMP cadets’ mental health challenges by offering the first known empirical evidence of mental health knowledge, stigma, and service use intentions in RCMP cadets starting the CTP. Consistent with the current study hypotheses and previous research (Hadlaczky et al., 2014; Schnyder et al., 2017; Xu et al., 2018; Krakauer et al., 2020), mental health knowledge and service use intentions were statistically significantly associated, and stigma was inversely statistically significantly associated with mental health knowledge and service use intentions. The results suggest that higher levels of mental health knowledge are associated with lower stigma and higher intention to use professional mental health services. The current results are novel and highlight the importance of research involving mental health training to increase mental health knowledge and service use intentions by reducing stigma around mental health disorders and help-seeking behaviors.

As hypothesized, cadets reported lower stigma and higher service use intentions than serving RCMP. However, unexpectedly cadets reported lower mental health knowledge compared to serving RCMP. The lower mental health knowledge and stigma observed among cadets as compared to serving members may be related to experience. Police officers are more likely than the general population to encounter people with a mental health disorder as first responders (Desmarais et al., 2014). Frequently attending to mental health emergencies while on-duty could likely increase perceived mental health knowledge. Additionally, experience dealing with people with mental health disorders may also increase an officer’s level of stigma toward those with mental health disorders. Compared to the general population, police officers have previously endorsed significantly more stigma, negative stereotypes, and perceptions of dangerousness and unpredictability toward those with mental health disorders (Soomro and Yanos, 2019). The current findings suggest that stigma may increase through experience. Mental health knowledge may also increase; however, the nature of the knowledge gained based on experiences is not clear. This knowledge could be biased if the experience was perceived as stressful and traumatic (Soomro and Yanos, 2019). Further research is needed to understand the associations between on-duty experience dealing with people with mental health disorders, perceived mental health knowledge based on these experiences and stigma.

Professional experience with mental health disorders may have also contributed to serving RCMP reporting lower service use intentions compared to cadets. Research with police suggests that police avoid using services in spaces where they bring civilians for mental health support (Newell et al., 2022). Given the rural nature of most RCMP postings, the only available mental health services in the area are likely to be places where officers have previously brought civilians for mental health services. Officers may not be aware of online resources available to them such as Internet-delivered cognitive behavioral therapy (McCall et al., 2021).

Overall, the differences in mental health knowledge, stigma, and service use intentions between cadets starting the CTP and serving RCMP suggest a need for initial (i.e., during the CTP) and ongoing (i.e., throughout an officer’s career) psychoeducation or evidence-informed training. Mental health training is likely to increase mental health knowledge, decrease stigma, and increase service use intentions in RCMP cadets and serving members, thereby protecting the mental health of RCMP members. There are now numerous options for such training (i.e., CISM, CISD, Peer support, BOS, R2MR, MHFA; Di Nota et al., 2021). The RCMP has been including R2MR training in the CTP since 2012. In addition, the Emotional Resilience Skills Training (ERST) currently being evaluated by the RCMP Study underscores education about mental health, which should implicitly reduce stigma (Carleton et al., 2022). Mental health knowledge has been associated with decreases in stigma and increases in service use intentions both in the general public (Hadlaczky et al., 2014; Schnyder et al., 2017; Xu et al., 2018) and in PSP (Krakauer et al., 2020). Future research is needed to determine if such mental health training is related to increased mental health knowledge, decreased stigma, increased service use intentions, and overall improved mental health.

Some differences between sociodemographic characteristics were also observed. As expected, female cadets reported statistically significantly higher mental health knowledge as well as statistically significantly higher service use intentions scores and statistically significantly lower stigma scores compared to male cadets. Greater mental health knowledge among females is consistent with previous PSP research (Krakauer et al., 2020). Also consistent with female PSP, female cadets have previously self-reported higher scores than males on symptom measures for PTSD, MDD, GAD, and SAD and were more likely to screen positive (Angehrn et al., 2022; Carleton et al., in press). As with female PSP, female cadets may be more likely to be aware of their mental health and more able to report their symptoms on a self-report questionnaire due to how females are socialized with in respect to mental health over the course of their lifetime (Merritt et al., 2014; Gibbons et al., 2015). Females are often socialized to express emotions and seek support when needed, which may develop their mental health knowledge more so than their male counterparts (Mankus et al., 2016). Increased mental health knowledge may increase the likelihood that a person can and will report difficulties (Krakauer et al., 2020). Additionally, males appear to be more likely to underreport symptoms of mental disorders (Berger et al., 2012) and may experience more stigma around reporting mental health difficulties and seeking support due to hypermasculine attitudes that are embedded in police culture (Berg et al., 2006). Further research is needed to understand how gender influences the relationship between mental health knowledge and mental health disorder symptom reporting among RCMP officers and cadets.

The differences in mental health knowledge and service use intentions observed between male and female cadets are further supported by male cadets reporting statistically significantly higher stigma. This was expected and is consistent with previous research reporting higher mental health stigma among male police officers (Soomro and Yanos, 2019). Stigma is a well-documented barrier to seeking support for mental health among PSP (Oliphant, 2016; Ricciardelli et al., 2018a,b; Krakauer et al., 2020; Carleton et al., 2020b; Newell et al., 2022). Stigma has been reported to deter officers from seeking help and from discussing distressing issues with other officers, out of fear of being deemed unfit for duty (Ricciardelli et al., 2018a). Police and communicators reported they felt stigma more oriented toward seeking support and less about experiencing mental health struggles (Newell et al., 2022). Both statistically significantly lower service use intentions and higher stigma were observed among male cadets compared to female cadets in the current results, further supporting stigma as a barrier to seeking support among male officers. Higher stigma, lower service use intentions, and lower mental health knowledge observed among male cadets in the current results suggest that male cadets may be less aware or able to recognize mental health difficulties and less likely to seek support when experiencing mental health challenges.

Increasing mental health knowledge and service use intentions and reducing stigma should begin at cadet training (Papazoglou and Andersen, 2014). Mental health training programs during the CTP are needed to inform cadets how to identify early signs associated with mental health challenges, occupational risk factors, barriers to seeking care including stigma, and provide cadets with self-care practices and information on mental health resources available to them (Carleton et al., 2019, 2022). Additionally, understanding how stigma might impact male and female officers can help to inform strategies and training that target these underlying factors. Overall, the current results suggest that cadets starting the CTP evidence levels of stigma lower than serving RCMP and service use intentions higher than serving RCMP and suggest that the differences arise from service experiences rather than individual factors present at recruitment. The current results also highlight the need for mental health training starting at the CTP and continuing throughout the career to increase awareness and normalization of mental health disorders and the benefits of officers having a working knowledge of mental health and mental health disorders while engaging with the public on-duty.

### 4.1. Strengths and limitations

The RCMP Study overall has several strengths and limitations (for details see Carleton et al., 2022). Strengths of the current study include the first empirical evidence on the mental health knowledge, stigma, and service use intentions of a large sample of RCMP cadets staring the CTP. The current study also highlights the magnitude of current mental health challenges of serving RCMP and a large representative sample of RCMP cadets. Limitations of the current study include: (1) data collection interruptions due to COVID-19, which resulted in data collection including participants from pre- and post-COVID-19 onset; (2) voluntary participation creating an unknowable influence from self-selection biases; (3) potential for socially desirable responding based on assessments occurring within the context of the CTP; (4) assessment of stigma toward others with mental disorders, but not self-stigma, which may influence an individual’s own service use intentions (Corrigan, 2004); and (5) a lower Cronbach’s Alpha observed for MAKS for the current sample compared to previously reported alphas for a diverse sample of PSP (Krakauer et al., 2020). The limitations may be offset by the sample size, the analyses, the participants’ ability to complete RCMP study tasks as part of paid time, the pre-registration of hypotheses, third party assessors, and participant anonymity. The sample size and the methods used to maintain confidentiality and anonymity of participants, also reduces the likelihood of socially desirable responding from participants.

## 5. Conclusion

RCMP Cadets starting CTP reported lower mental health knowledge and stigma, and higher mental health services use intentions compared to serving RCMP. The results indicate that higher levels of mental health knowledge were associated with lower stigma and higher intention to use professional mental health services. The findings highlight the importance of mental health training which may be useful for improving mental health knowledge and, in turn, decreasing stigma and increasing mental health service use intentions. Future research is needed to determine if such mental health training is related to increased mental health knowledge, decreased stigma, increased service use intentions, and overall improved mental health.

Differences in mental health knowledge, stigma, and service use intentions were observed between male and female cadets. Stigma may be a barrier to seeking support among male cadets. To better understand the underlying sex differences, further research involving both qualitative and quantitative methods should be conducted to assess how sex may influence mental health in the RCMP. The results provide a baseline to improve cadet mental health knowledge and service use intentions and reduce stigma with further mental health training.

## Data availability statement

The datasets presented in this article are not readily available due to the sensitive nature of the content and to protect anonymity of participants. Requests to access the datasets should be directed to katie.andrews@uregina.ca.

## Ethics statement

The studies involving human participants were reviewed and approved by University of Regina Institutional Research Ethics Board. The participants provided their written informed consent to participate in this study.

## Author contributions

KA, RC, TA, AF, RS, and AB: conceptualization. KA, RC, TA, LJ, and RS: methodology. KA, RC, LJ, AF, TA, and AB: validation. KA, RC, LJ, TT, and TA: formal analyses. KA, LJ, RC, AB, GK, and SS-Z: investigation. RC, AB, GK, and TA: resources. KA, RC, TT, LJ, RS, and AB: data curation. KA, RC, AF, LJ, TT, TA, and RS: writing—original draft preparation. KA, LJ, RS, TT, TA, AF, SS-Z, AB, GK, and RC: writing—review and editing. RC and GK: Supervision. KA, RC, GK, and TA: project administration. NC, AF, GK, and SS-Z: funding acquisition. All authors viewed and approved the submitted version of the manuscript.

## Funding

The RCMP Study is supported by the RCMP, the Government of Canada, and the Ministry of Public Safety and Emergency Preparedness. TA is supported by a Tier I Canada Research Chair in Childhood Adversity and Resilience. The development, analyses, and distribution of the current article were supported by a generous grant from the Medavie Foundation.

## Conflict of interest

The authors declare that the research was conducted in the absence of any commercial or financial relationships that could be construed as a potential conflict of interest.

## Publisher’s note

All claims expressed in this article are solely those of the authors and do not necessarily represent those of their affiliated organizations, or those of the publisher, the editors and the reviewers. Any product that may be evaluated in this article, or claim that may be made by its manufacturer, is not guaranteed or endorsed by the publisher.

## References

[ref1] Anderson G. S. Di Nota P. M. Groll D. Carleton R. N. (2020). Peer support and crisis-focused psychological interventions designed to mitigate post-traumatic stress injuries among public safety and frontline healthcare personnel: a systematic review. Int. J. Environ. Res. Public Health 17:7645. doi: 10.3390/ijerph17207645, PMID: 33092146 PMC7589693

[ref2] Angehrn A. Vig K. D. Mason J. E. Stelnicki A. M. Shields R. E. Asmundson G. J.. (2022). Sex differences in mental disorder symptoms among Canadian police officers: the mediating role of social support, stress, and sleep quality. Cogn. Behav. Ther. 51, 3–20. doi: 10.1080/16506073.2021.1877338, PMID: 33554743

[ref3] Berg A. M. Hem E. Lau B. R. Ekeberg Ø. (2006). Help-seeking in the Norwegian police service. J. Occup. Health 48, 145–153. doi: 10.1539/joh.48.145, PMID: 16788274

[ref66] Berger J. L. Addis M. E. Reilly E. D. Syzdek M. R. Green J. D. (2012). Effects of gender, diagnostic labels, and causal theories on willingness to report symptoms of depression. J. Soc. Clin. Psychol. 31, 439–457.

[ref4] Beshai S. Carleton R. N.. (2016). Peer support and crisis-focused psychological intervention programs in Canadian first responders: blue paper; University of Regina Collaborative Centre for Justice and Safety: Regina, SK, Canada, Available at:: http://www.justiceandsafety.ca/rsu_docs/blue_paper_full_web_final_production_aug_16_2016.pdf (Accessed October 19, 2020).

[ref5] Blum L. N. (2000). Force under pressure: how cops live and why they die. Lagos, Nigeria: Lantern Books.

[ref6] Boehme B. A. Shields R. E. Asmundson G. J. Szeto A. C. Dobson K. S. Carleton R. N. (2022). A short version of the Opening Minds Scale -Workplace Attitudes: Factor structure and factorial validity in a sample of Canadian public safety personnel. Can. J. Behav. Sci.

[ref7] Canadian Institute for Public Safety Research and Treatment (CIPSRT) (2019). Glossary of terms: a shared understanding of the common terms used to describe psychological trauma (version 2.1). Regina, SK: Author.

[ref8] Carleton R. N. Afifi T. O. Taillieu T. Turner S. Krakauer R. Anderson G. S.. (2019). Exposures to potentially traumatic events among public safety personnel in Canada. Can. J. Behav. Sci. 51, 37–52. doi: 10.1037/cbs0000115

[ref9] Carleton R. N. Afifi T. O. Taillieu T. Turner S. Mason J. E. Ricciardelli R.. (2020a). Assessing the relative impact of diverse stressors among public safety personnel. Int. J. Environ. Res. Public Health 17:1234. doi: 10.3390/ijerph17041234, PMID: 32075062 PMC7068554

[ref10] Carleton R. N. Afifi T. O. Turner S. Taillieu T. Duranceau S. DM L. B.. (2018a). Mental disorder symptoms among public safety personnel in Canada. Can. J. Psychiatry 63, 54–64. doi: 10.1177/0706743717723825, PMID: 28845686 PMC5788123

[ref11] Carleton R. N. Afifi T. O. Turner S. Taillieu T. Vaughan A. D. Anderson G. S.. (2020b). Mental health training, attitudes towards support, and screening positive for mental disorders. Cogn. Behav. Ther. 49, 55–73. doi: 10.1080/16506073.2019.1575900, PMID: 30794073

[ref12] Carleton R. N. Jamshidi L. Maguire K. Lix L. Stewart S. Afifi T.. (in press). Mental health of Royal Canadian Mounted Police at the start of the cadet training program. Can. J. Psychiatry/La Rev. Can. Psychiatr. 10.1177/07067437221147425 PMC10585131 37131322

[ref13] Carleton R. N. Korol S. Mason J. E. Hozempa K. Anderson G. S. Jones N. A.. (2018b). A longitudinal assessment of the road to mental readiness training among municipal police. Cogn. Behav. Ther. 47, 508–528. doi: 10.1080/16506073.2018.1475504, PMID: 29912631

[ref14] Carleton R. N. Krätzig G. P. Sauer-Zavala S. Neary J. P. Lix L. M. Fletcher A. J.. (2022). Study protocol–the Royal Canadian Mounted Police (RCMP) study: protocol for a prospective investigation of mental health risk and resilience factors. Health Promot. Chron 42, 319–333. doi: 10.24095/hpcdp.42.8.02, PMID: 35993603 PMC9514212

[ref15] Cheng H. L. Wang C. McDermott R. C. Kridel M. Rislin J. L. (2018). Self-stigma, mental health literacy, and attitudes toward seeking psychological help. J. Couns. Dev. 96, 64–74. doi: 10.1002/jcad.12178

[ref16] Clement S. Schauman O. Graham T. Maggioni F. Evans-Lacko S. Bezborodovs N.. (2015). What is the impact of mental health-related stigma on help-seeking? A systematic review of quantitative and qualitative studies. Psychol. Med. 45, 11–27. doi: 10.1017/S0033291714000129, PMID: 24569086

[ref17] Conner K. O. Copeland V. C. Grote N. K. Koeske G. Rosen D. Reynolds C. F. III. (2010). Mental health treatment seeking among older adults with depression: the impact of stigma and race. Am. J. Geriatr. Psychiatry 18, 531–543. doi: 10.1097/JGP.0b013e3181cc0366, PMID: 20220602 PMC2875324

[ref18] Corrigan P. (2004). How stigma interferes with mental health care. Am. Psychol. 59, 614–625. doi: 10.1037/0003-066X.59.7.614 15491256

[ref19] Desmarais S. L. Livingston J. D. Greaves C. L. Johnson K. L. Verdun-Jones S. Parent R.. (2014). Police perceptions and contact among people with mental illnesses: comparisons with a general population survey. Psychol. Public Policy Law 20, 431–442. doi: 10.1037/law0000023

[ref20] Di Nota P. M. Bahji A. Groll D. Carleton R. N. Anderson G. S. (2021). Proactive psychological programs designed to mitigate posttraumatic stress injuries among at-risk workers: a systematic review and meta-analysis. BMC Syst. Rev. 10:126. doi: 10.1186/s13643-021-01677-7, PMID: 33910641 PMC8079856

[ref190] Evans-Lacko S. Little K. Meltzer H. Rose D. Rhydderch D. Henderson C.. (2010). Development and psychometric properties of the mental health knowledge schedule. Can. J. Psychiatry 55, 440–448. 20704771 10.1177/070674371005500707

[ref21] Fikretoglu D. Liu A. Nazarov A. Blackler K. (2019). A group randomized control trial to test the efficacy of the road to mental readiness (r2mr) program among Canadian military recruits. BMC Psychiatry 19:326. doi: 10.1186/s12888-019-2287-0, PMID: 31664960 PMC6819517

[ref212] Gibbons R. J. Thorsteinsson E. B. Loi N. M. (2015). Beliefs and attitudes towards mental illness: an examination of the sex differences in mental health literacy in a community sample. PeerJ 3:e1004. 26413429 10.7717/peerj.1004 PMC4581769

[ref22] Hadlaczky G. Hökby S. Mkrtchian A. Carli V. Wasserman D. (2014). Mental health first aid is an effective public health intervention for improving knowledge, attitudes, and behaviour: a meta-analysis. Int. Rev. Psychiatry 26, 467–475. doi: 10.3109/09540261.2014.924910, PMID: 25137113

[ref23] Hembroff C. C. Krätzig G. (2020). “A 5-year perspective of attrition in relation to employment equity,” in RCMP Depot Division: Training. (Innovation and Research).

[ref24] Jorm A. F. (2012). Mental health literacy: empowering the community to take action for better mental health. Am. Psychol. 67, 231–243. doi: 10.1037/a0025957, PMID: 22040221

[ref25] Karaffa K. M. Koch J. M. (2016). Stigma, pluralistic ignorance, and attitudes toward seeking mental health services among police officers. Crim. Justice Behav. 43, 759–777. doi: 10.1177/0093854815613103

[ref26] Kirschman E. Kamena M. Fay J. (2015). Counseling cops: what clinicians need to know. New York, NY: Guilford Publications.

[ref27] Klimley K. E. Van Hasselt V. B. Stripling A. M. (2018). Posttraumatic stress disorder in police, firefighters, and emergency dispatchers. Aggress. Violent Behav. 43, 33–44. doi: 10.1016/j.avb.2018.08.005

[ref28] Krakauer R. L. Stelnicki A. M. Carleton R. N. (2020). Examining mental health knowledge, stigma, and service use intentions among public safety personnel. Front. Psychol. 11: 949. doi: 10.3389/fpsyg.2020.00949, PMID: 32547443 PMC7273931

[ref30] Levy-Gigi E. Richter-Levin G. Kéri S. (2014). The hidden price of repeated traumatic exposure: different cognitive deficits in different first-responders. Front. Behav. Neurosci. 8:281. doi: 10.3389/fnbeh.2014.00281 25191237 PMC4138485

[ref310] Mankus A. M. Boden M. T. Thompson R. J. (2016). Sources of variation in emotional awareness: Age, gender, and socioeconomic status. Person. Individ. Differ. 89, 28–33. doi: 10.1016/j.paid.2015.09.043 PMC4612349 26500384

[ref31] Maslowski A. K. LaCaille R. A. LaCaille L. J. Reich C. M. Klingner J. (2019). Effectiveness of mental health first aid: a meta-analysis. Ment. Health Rev. J. 24, 245–261. doi: 10.1108/MHRJ-05-2019-0016

[ref32] McCall H. C. Beahm J. D. Fournier A. K. Burnett J. L. Carleton R. N. Hadjistavropoulos H. D. (2021). Stakeholder perspectives on internet-delivered cognitive behavioural therapy for public safety personnel: a qualitative analysis. Can J. Behav. Sci./Revue canadienne des sciences du comportement 53, 232–242. doi: 10.1037/cbs0000242

[ref311] Merritt C. J. Tharp I. J. Furnham A. (2014). Trauma type affects recognition of Post-Traumatic Stress Disorder among online respondents in the UK and Ireland. J. affect. disor. 164, 123–129. 10.1016/j.jad.2014.04.013 24856565

[ref33] Newell C. J. Ricciardelli R. Czarnuch S. M. Martin K. (2022). Police staff and mental health: barriers and recommendations for improving help-seeking. Police Pract. Res. 23, 111–124. doi: 10.1080/15614263.2021.1979398

[ref34] Oliphant R. C. (2016). Healthy minds, safe communities: supporting our public safety officers through a National Strategy for operational stress injuries; security, standing committee on public safety and National Security, Standing Committee on Public Safety and National Security: Ottawa, ON, Canada.

[ref35] Papazoglou K. Andersen J. P. (2014). A guide to utilizing police training as a tool to promote resilience and improve health outcomes among police officers. Traumatol. Int. J. 20, 103–111. doi: 10.1037/h0099394

[ref36] Paton D. Violanti J. Schmuckler E. (1999). Chronic exposure to risk and trauma: addiction and separation issues in police officers. Police trauma: Psychological aftermath of civilian combat. 78–87.

[ref37] Price J. A. Landry C. A. Sych J. McNeill M. Stelnicki A. M. Asmundson A. J.. (2022). Assessing the perceptions and impact of critical incident stress management peer support among firefighters and paramedics in Canada. Int. J. Environ. Res. Pub Hea. 19:4976. doi: 10.3390/ijerph19094976, PMID: 35564374 PMC9100761

[ref38] Public Safety Canada (2019). “Supporting canada's public safety personnel: an action plan on post-traumatic stress injuries” in Preparedness PSAE (Ottawa, ON: Government of Canada)

[ref39] Reavley N. J. Jorm A. F. (2011). Recognition of mental disorders and beliefs about treatment and outcome: findings from an Australian national survey of mental health literacy and stigma. Aust. N.Z. J. Psychiatry 45, 947–956. doi: 10.3109/00048674.2011.621060, PMID: 21995330

[ref40] Ricciardelli R. Carleton R. N. Groll D. Cramm H. (2018a). Qualitatively unpacking Canadian public safety personnel experiences of trauma and their well-being. Can. J. Criminol. Crim. 60, 566–577. doi: 10.3138/cjccj.2017-0053.r2

[ref41] Ricciardelli R. Carleton R. N. Mooney T. Cramm H. (2018b). “Playing the system”: structural factors potentiating mental health stigma, challenging awareness, and creating barriers to care for Canadian public safety personnel. Health 2018, 259–278. doi: 10.1177/136345931880016 32283964

[ref43] Schnyder N. Panczak R. Groth N. Schultze-Lutter F. (2017). Association between mental health-related stigma and active help-seeking: systematic review and meta-analysis. Br. J. Psychiatry 210, 261–268. doi: 10.1192/bjp.bp.116.189464, PMID: 28153928

[ref44] Sherwood L. Hegarty S. Vallières F. Hyland P. Murphy J. Fitzgerald G.. (2019). Identifying the key risk factors for adverse psychological outcomes among police officers: a systematic literature review. J. Trauma. Stress. 32, 688–700. doi: 10.1002/jts.22431, PMID: 31553502

[ref45] Skogstad M. Skorstad M. Lie A. Conradi H. S. Heir T. Weisaeth L. (2013). Work-related post-traumatic stress disorder. Occup. Med. 63, 175–182. doi: 10.1093/occmed/kqt003 23564090

[ref46] Soomro S. Yanos P. T. (2019). Predictors of mental health stigma among police officers: the role of trauma and PTSD. J. Police Crim. Psychol. 34, 175–183. doi: 10.1007/s11896-018-9285-x

[ref47] Statistics Canada (2014). Table: 13-10-0465-01 (formerly cansim 105–1101). Ottawa, ON, Canada: Government of Canada.

[ref48] Szeto A. Dobson K. S. Knaak S. (2019). The road to mental readiness for first responders: a meta-analysis of program outcomes. Can. J. Psychiatr. 64, 18S–29S. doi: 10.1177/0706743719842562 PMC6591743 31010293

[ref49] Vaughan A. D. Stoliker B. E. Anderson G. S. (2020). Building personal resilience in primary care paramedic students, and subsequent skill decay. Australas J. Paramed. 17, 1–8. doi: 10.33151/ajp.17.803

[ref50] Violanti J. M. (2006). The police: perspectives on trauma and resiliency. Traumatology 12, 167–169. doi: 10.1177/1534765606296998

[ref51] Violanti J. M. (2010). Police suicide: a national comparison with fire-fighter and military personnel. Policing Inter. J. Police Strat. Manag. 33, 270–286. doi: 10.1108/13639511011044885

[ref52] Violanti J. M. (2015). “Of mind and body: health consequences of stress and trauma on police officers,” in Behind the badge: A psychological treatment handbook for law enforcement officers. eds. Clevenger S. M. F. Miller L. Moore B. A. Freeman A. (Routledge/Taylor & Francis Group), 44–69.

[ref54] WGM Psychological Services (2018). Before operational stress: BOS training manual for certified clinicians. Calgary, AB:WGM Psychological Services.

[ref55] Wheeler C. Fisher A. Jamiel A. Lynn T. J. Hill W. T. (2021). Stigmatizing attitudes toward police officers seeking psychological services. J. Police Crim. Psychol. 36, 1–7. doi: 10.1007/s11896-018-9293-x

[ref56] White A. K. Shrader G. Chamberlain J. (2016). Perceptions of law enforcement officers in seeking mental health treatment in a right-to-work state. J. Police Crim. Psychol. 31, 141–154. doi: 10.1007/s11896-015-9175-4

[ref58] Wilmoth J. A. (2014). Trouble in mind. National Fire Protection Association (NFPA) J.

[ref59] Woody R. H. (2006). Family interventions with law enforcement officers. Am. J. Fam. Ther. 34, 95–103. doi: 10.1080/01926180500376735

[ref60] Xu Z. Huang F. Koesters M. Staiger T. Becker T. Thornicroft G.. (2018). Effectiveness of interventions to promote help-seeking for mental health problems: systematic review and meta-analysis. Psychol. Med. 48, 2658–2667. doi: 10.1017/S0033291718001265 29852885

